# Tachycardia in a patient with mild traumatic brain injury

**DOI:** 10.1007/s10286-019-00646-4

**Published:** 2019-10-21

**Authors:** Sung Ho Jang, Young Hyeon Kwon, Sung Jun Lee

**Affiliations:** Department of Physical Medicine and Rehabilitation, College of Medicine, Yeungnam University, 317-1, Daemyungdong, Namku, Taegu, 705-717 Republic of Korea

**Keywords:** Mild traumatic brain injury, Tachycardia, Paroxysmal sympathetic hyperactivity, Head trauma, Brain injury

Dear editors

Traumatic brain injury (TBI) is classified as mild, moderate, or severe, and mild TBI accounts for 70–90% of all TBI [1]. Tachycardia following TBI is a sign of paroxysmal sympathetic hyperactivity (PSH), which consists of fever, hypertension (systolic blood pressure > 160 mmHg), tachypnea (respiratory rate > 30 breaths/minute), excessive diaphoresis, and extensor posturing or severe dystonia [2, 3]. PSH has been reported mainly in patients with severe TBI and diffuse axonal injury [4–6]. However, very little has been reported on PSH in mild TBI. In this case report, we report on a patient who displayed tachycardia following mild TBI.

A 25-year-old male patient suffered head trauma resulting from a motor vehicle accident. While he was riding downslope on a bicycle, he collided with a bus and fell to the ground. He reported that he lost consciousness for approximately several minutes after the fall and experienced post-traumatic amnesia for approximately one hour. The patient’s Glasgow Coma Scale score was 15 when he arrived at the hospital. Conventional brain MRI did not detect any abnormality (Fig. 1a). Following the accident, he began to experience tachycardia. During tachycardia, his pulse rate has increased to a maximum of 171 beats/minute, and his systolic blood pressure has reached 140 ~ 155 mmHg. He reported that the tachycardia was aggravated with postural change (from lying to standing) and running. When he visited the cardiology department of a university hospital at three weeks after onset, his pulse rate was 115 beats/minute on electrocardiography (Fig. 1b). The echocardiography and cardiac enzyme studies (troponin I: 0.01 ng/ml (0 ~ 0.04), and CK-MB: 2 ng/ml (0.6 ~ 6.3) did not show any abnormality. Moreover, he had no history of cardiac disease, medical or neurological conditions, or previous TBI. He also reported no family history of cardiac disease. He provided signed, informed consent to participate in this study, and the study protocol was approved by the institutional review board of our university hospital.

**Fig. 1:**
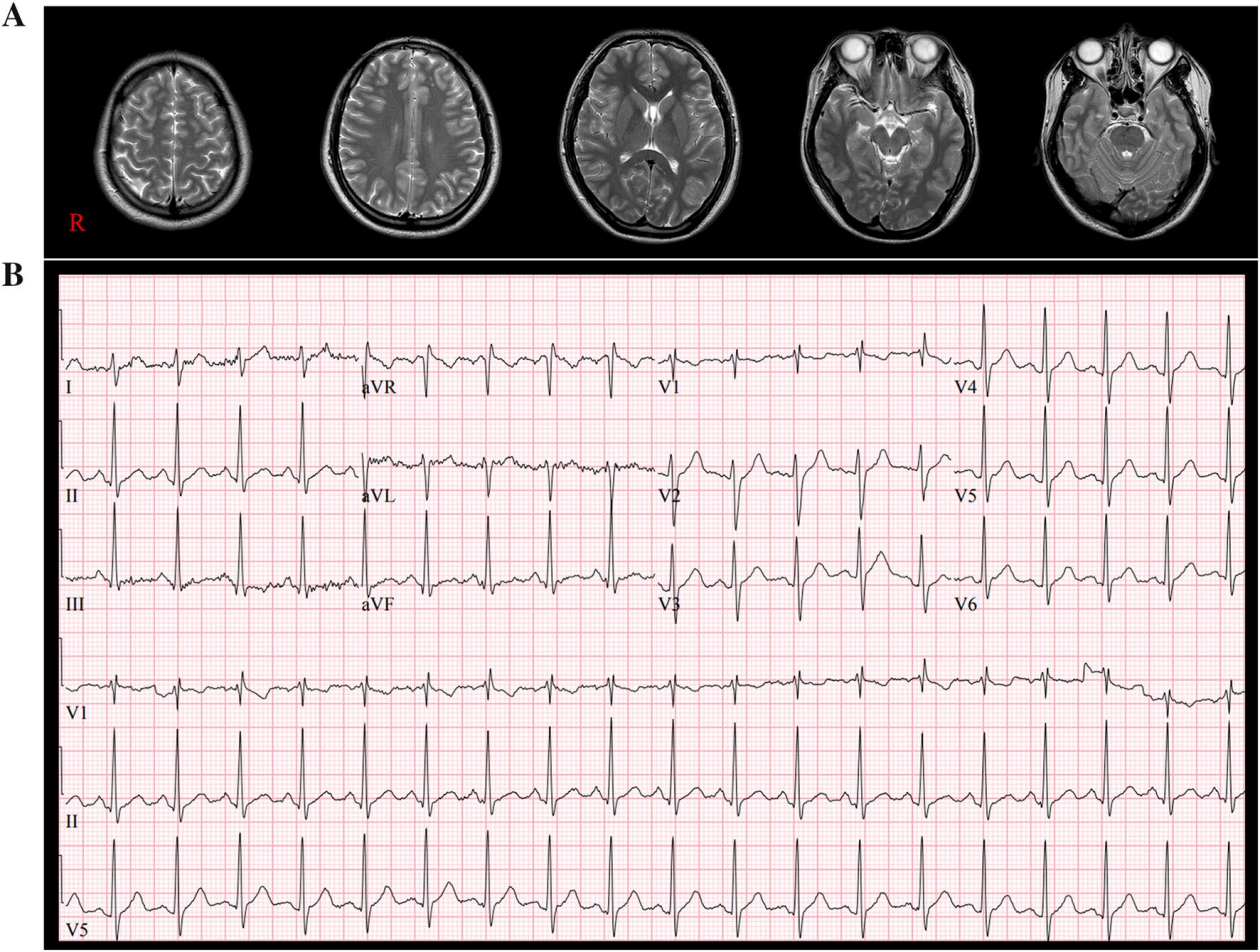
**a** T2-weighted brain magnetic resonance images at five months after onset show no abnormality. **b** Electrocardiography at three weeks after onset showing presence of tachycardia at 115 beats/minute

In this case report, we report on a patient who showed tachycardia (maximum heart rate recorded was 171 beats/minute) and mild hypertension following a fall resulting in mild TBI. Although tachycardia is the only sign corresponding to the criteria of PSH, the presence of tachycardia and mild hypertension in this patient were considered signs of sympathetic hyperactivity following brain injury due to head trauma [2].

The pathophysiological mechanisms of PSH remain unclear. However, two main mechanisms have been suggested: (1) simple disconnection of cortical inhibitory centers such as the insula and cingulate cortex to the brain areas which are responsible for supraspinal control of sympathetic tone (hypothalamus, diencephalon, and brainstem), (2) the excitatory:inhibitory ratio model; paroxysms are driven by abnormal processing of afferent stimuli within the spinal cord following disconnection of descending inhibitory pathways [7–9]. Although the patient’s conventional brain MRI results did not reveal any abnormality, we speculate that TBI, which cannot be detected on conventional brain MRI, might be the plausible pathophysiologic mechanism of tachycardia in this patient [8].

In conclusion, we describe a patient who showed tachycardia following mild TBI. To our knowledge, this patient is the first reported case of tachycardia following mild TBI. Further studies involving a larger number of subjects showing signs of sympathetic hyperactivity following mild TBI should be undertaken. In addition, further studies to elucidate the pathophysiology associated with signs of sympathetic hyperactivity following mild TBI should be encouraged.

## References

[1] Mild Traumatic Brain Injury Committee (1993). Definition of mild traumatic brain injury. J Head Trauma Rehabil.

[2] Rabinstein AA (2007). Paroxysmal sympathetic hyperactivity in the neurological intensive care unit. Neurol Res.

[3] Perkes IE, Menon DK, Nott MT, Baguley IJ (2011). Paroxysmal sympathetic hyperactivity after acquired brain injury: a review of diagnostic criteria. Brain Inj.

[4] Baguley IJ, Nicholls JL, Felmingham KL, Crooks J, Gurka JA, Wade LD (1999). Dysautonomia after traumatic brain injury: a forgotten syndrome?. J Neurol Neurosurg Psychiatry.

[5] Fernandez-Ortega JF, Prieto-Palomino MA, Garcia-Caballero M, Galeas-Lopez JL, Quesada-Garcia G, Baguley IJ (2012). Paroxysmal sympathetic hyperactivity after traumatic brain injury: clinical and prognostic implications. J Neurotrauma.

[6] Deepika A, Mathew MJ, Kumar SA, Devi BI, Shukla D (2015). Paroxysmal sympathetic hyperactivity in pediatric traumatic brain injury: a case series of four patients. Auton Neurosci.

[7] Baguley IJ, Heriseanu RE, Cameron ID, Nott MT, Slewa-Younan S (2008). A critical review of the pathophysiology of dysautonomia following traumatic brain injury. Neurocrit Care.

[8] Meyfroidt G, Baguley IJ, Menon DK (2017). Paroxysmal sympathetic hyperactivity: the storm after acute brain injury. Lancet Neurol.

[9] Godoy DA, Panhke P, Guerrero Suarez PD, Murillo-Cabezas F (2019). Paroxysmal sympathetic hyperactivity: an entity to keep in mind. Med Intensiva.

